# Supplementary material for: TraceTrack, an open-source software for batch processing, alignment and visualization of sanger sequencing chromatograms
Source: Bioinform Adv. 2023 Jul 12;3(1):vbad083. doi: 10.1093/bioadv/vbad083 (PMC10348866; doi:10.1093/bioadv/vbad083)
Supplement: vbad083_Supplementary_Data [file vbad083_supplementary_data.docx]

**TraceTrack, an Open-Source Software for Batch Processing, Alignment and Visualization of Sanger Sequencing Chromatograms**

Kveta Brazdilova, David Prihoda, Quynh Ton, Heath Klock and Danny A. Bitton

**Supplementary Material**


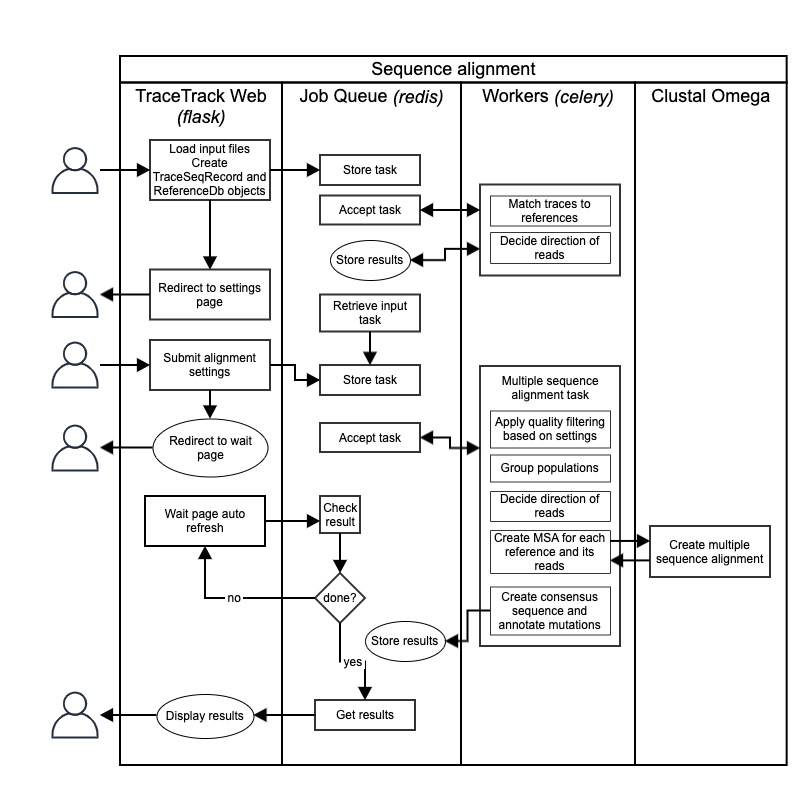


**Supplementary Figure 1**. Tool architecture. In the basic workflow, the user uploads a set of trace files and a sheet containing reference sequences, which are queued as a task. Next, the user inputs settings, which are stored as another task. This task is accepted from the queue by the worker and corresponding objects are created from trace and reference sequences. These are then quality trimmed, grouped and reversed where necessary, before the resulting multiple sequence alignment is produced for each reference sequence with its respective traces. The result is then stored. The frontend checks for the result regularly and when it is available, displays it to the user.


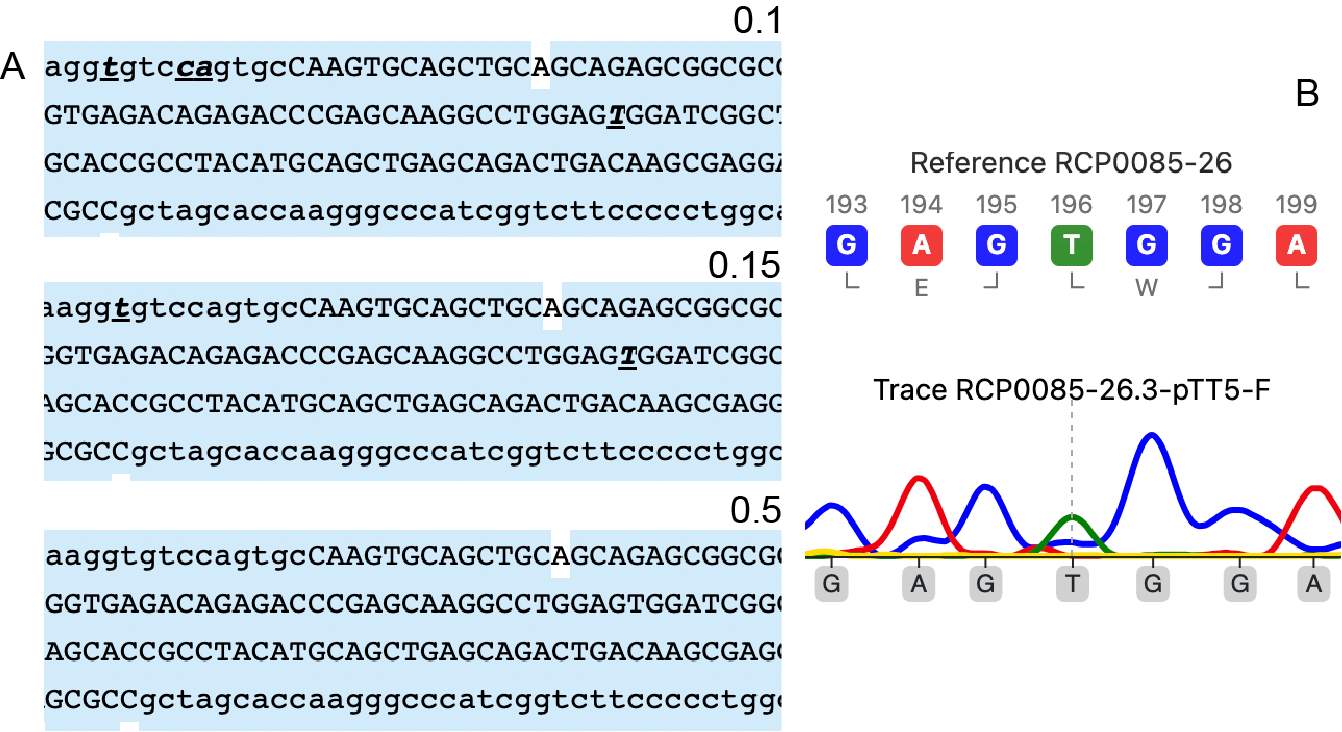


**Supplementary Figure 2**. Mixed peak detection. (A) Sensitivity of mixed peak detection changes with the parameter *f*. Detected mixed peaks (highlighted in the alignments) are shown for three values of *f*. (B) Screenshot of trace viewer centred around a detected mixed peak.

**Supplementary Table 1.** Versions of packages used for the deployment of the TraceTrack application.

_libgcc_mutex=0.1

_openmp_mutex=4.5

amqp=5.1.1

argtable2=2.13

async-timeout=4.0.2

attrs=21.4.0

backports=1.0

backports.functools_lru_cache=1.6.4

billiard=3.6.4.0

biopython=1.79

ca-certificates=2022.6.15

cached-property=1.5.2

cached_property=1.5.2

celery=5.2.7

click=8.1.3

click-didyoumean=0.3.0

click-plugins=1.1.1

click-repl=0.2.0

clustalo=1.2.4=

deprecated=1.2.13

et_xmlfile=1.0.1

flask=2.1.3

flower=1.1.0

gunicorn=20.1.0

humanize=4.2.3

icu=69.1

importlib-metadata=4.11.4

importlib_metadata=4.11.4

iniconfig=1.1.1

itsdangerous=2.1.2

jansson=2.14

jinja2=3.1.2

kombu=5.2.4

ld_impl_linux-64=2.36.1

libblas=3.9.0

libcblas=3.9.0

libffi=3.4.2

libgcc-ng=12.1.0

libgfortran-ng=12.1.0

libgfortran5=12.1.0

libgomp=12.1.0

libiconv=1.16

liblapack=3.9.0

libnsl=2.0.0

libopenblas=0.3.20

libstdcxx-ng=12.1.0

libxml2=2.9.12

libzlib=1.2.12

markupsafe=2.1.1

ncurses=6.3

numpy=1.21.6

openpyxl=3.0.9

openssl=3.0.5

packaging=21.3

pandas=1.3.5

parasail-python=1.2.4

pcre=8.45

pip=22.1.2

pluggy=1.0.0

prometheus_client=0.14.1

prompt-toolkit=3.0.30

prompt_toolkit=3.0.30

py=1.11.0

pyparsing=3.0.9

pytest=7.1.2

pytest-mock=3.8.2

python=3.7.12

python-dateutil=2.8.2

python_abi=3.7

pytz=2022.1

pyyaml=6.0

readline=8.1.2

redis-py=4.3.4

setuptools=59.8.0

six=1.16.0

sqlite=3.39.2

tk=8.6.12

tomli=2.0.1

tornado=6.2

typing-extensions=4.3.0

typing_extensions=4.3.0

uwsgi=2.0.20

vine=5.0.0

wcwidth=0.2.5

werkzeug=2.2.0

wheel=0.37.1

wrapt=1.14.1

xlsxwriter=3.0.3

xz=5.2.5

yaml=0.2.5

zipp=3.8.0

zlib=1.2.12

tracetrack==0.0.0

**Supplementary Table 2.** Comparison between TraceTrack and freely available and commercial tools. Note that commercial tools were not tested but rather evaluated from the feature list published on their respective websites.

| Tool | TraceTrack | Tracy | Sage | Pearl | Teal | sangeranalyseR | Geneious Prime | Snap gene | Laseregene Genomics |
| --- | --- | --- | --- | --- | --- | --- | --- | --- | --- |
| Alignment of single ab1 file | ✔ | ✔ | ✔ | ✔ | NA | ✔ | ✔ | ✔ | ✔ |
| Alignment of multiple ab1 files | ✔ |  |  | ✔ | NA | ✔ | ✔ | ✔ | ✔ |
| Alignment of Fwd and Rev files | ✔ | ✔ |  | ✔ | NA | ✔ | ✔ | ✔ | ✔ |
| Alignment to reference sequence | ✔ | ✔ | ✔ | ✔ | NA |  | ✔ | ✔ | ✔ |
| Alignment to multiple references (automatic matching) | ✔ |  |  |  | NA |  | ✔ |  | ✔ |
| Shows translation | ✔ |  |  |  | NA | ✔ | ✔ | ✔ | ✔ |
| Highlight region of interest (capitals) | ✔ | NA |  |  |  |  |  | ✔ |  |
| Integrated visualization of chromatogram | ✔ | NA | ✔ | ✔ | ✔ | ✔ | ✔ | ✔ | ✔ |
| Display full alignment with highlighted mutations | ✔ | NA | ✔ | ✔ | NA | ✔ |  |  |  |
| Open source | ✔ | ✔ | ✔ | ✔ | ✔ | ✔ |  |  |  |
| Integrated GUI | ✔ |  | ✔ | ✔ | ✔ | ✔ | ✔ | ✔ | ✔ |
| Export | ✔ | NA |  | ✔ | NA | ✔ | ✔ | ✔ | ✔ |

**Supplementary Table 3.** Performance of the TraceTrack application. File size was approximately 220 KB in all cases.

|  | 5 files  Aligned to different references | 5 files  Aligned to the same reference | 100 files  Aligned to different references | 100 files  Aligned to the same reference |
| --- | --- | --- | --- | --- |
| Running locally (MacBook pro, 32 GB RAM) | 6s | 5s | 13s | 25s |
| Running on the server | 8s | 6s | 91s | 42s |
